# Supplementary material for: Super-Low Friction Electrification Achieved on Polytetrafluoroethylene Films-Based Triboelectric Nanogenerators Lubricated by Graphene-Doped Silicone Oil
Source: Micromachines (Basel). 2023 Sep 16;14(9):1776. doi: 10.3390/mi14091776 (PMC10536030; doi:10.3390/mi14091776)
Supplement: Supplementary file 1 [file micromachines-14-01776-s001.zip › Supplementary Material-2594572-rev.pdf]

Supplementary Material

Super-Low Friction Electrification Achieved on Polytetrafluoroethylene Films-Based Triboelectric Nanogenerators Lubricated by Graphene-Doped Silicone Oil

Junzhao Chen [†], Yu Zhao [†], Ruirui Wang and Pengfei Wang *

Institute of Nanosurface Science and Engineering (INSE), State Key Laboratory of Radio Frequency Heterogeneous Integration, College of Mechatronics and Control Engineering, Shenzhen University, Shenzhen 518060, China

* Corresponding author. E-mail address: wangpf@szu.edu.cn (P. W.)

[†] These authors contributed equally to this work

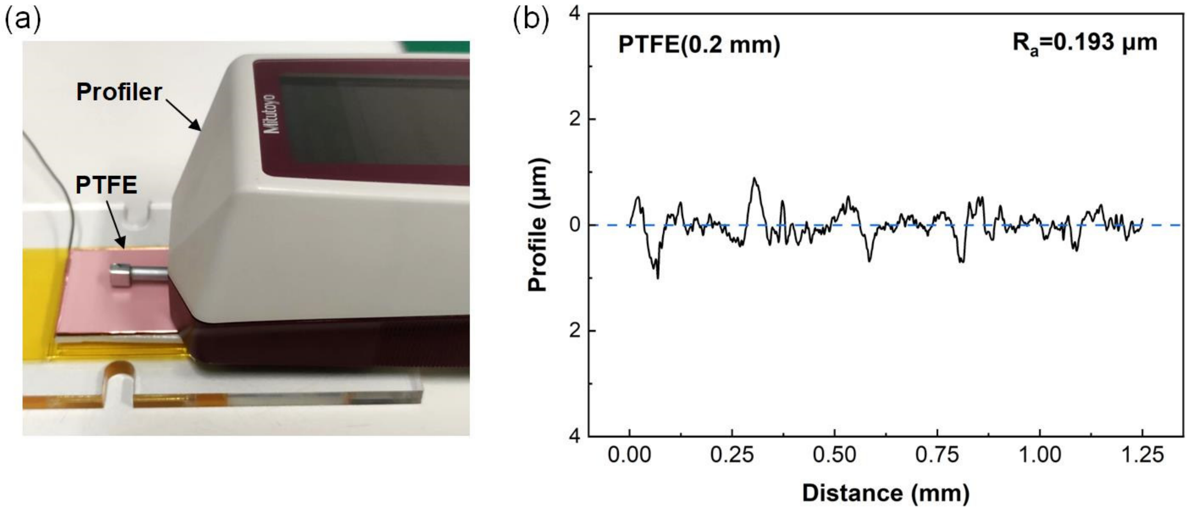

Figure S1. Measurement of surface roughness of the pristine PTFE film. (a) Photo of the surface profiler and the pristine PTFE film. (b) Typical surface profile of the pristine PTFE film.

Table S1. Surface roughness of the pristine PTFE film (thickness of 0.2 mm).

No.	R_a (μm)
1	0.194
2	0.181
3	0.190
4	0.193
5	0.208

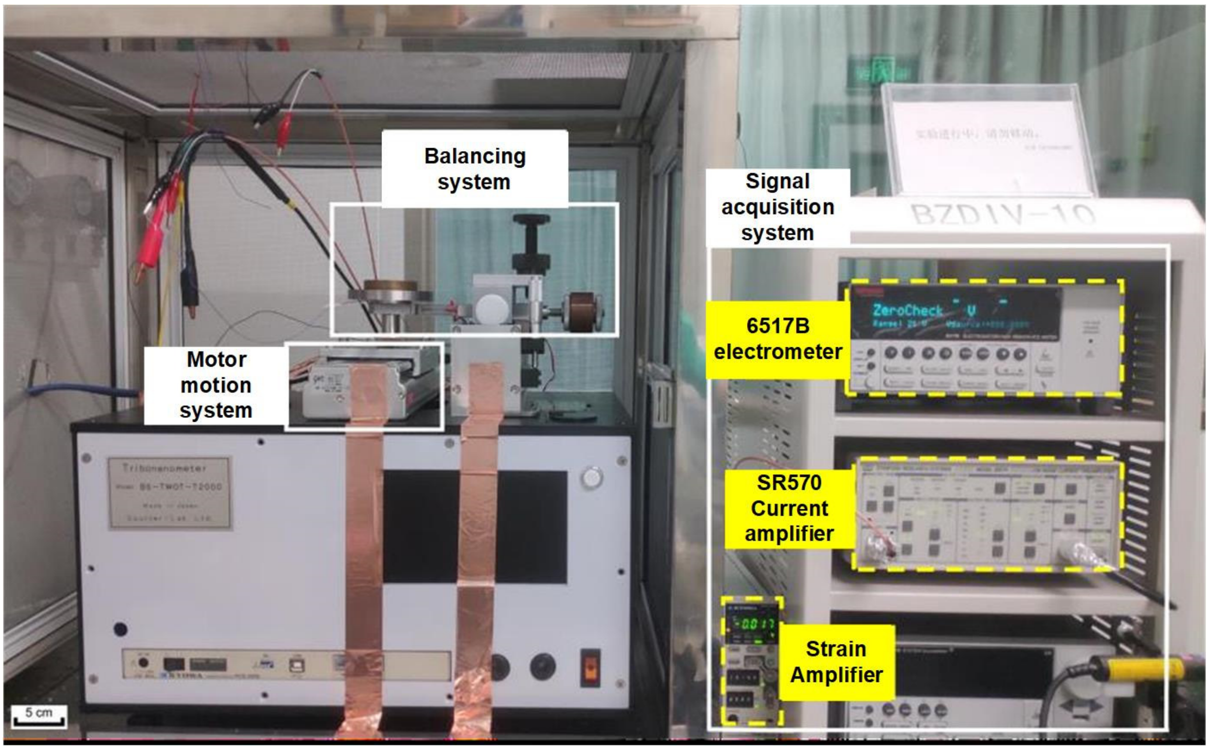

Figure S2. Photograph of the customized testing platform containing a ball-on-flat reciprocating sliding tribometer which could simultaneously measure the friction coefficient, short-circuit current, and open-circuit voltage of the PTFE films under dry friction and liquid lubrication conditions.

Table S2. Main properties of different liquid lubricants.

Lubricants	Description	Dynamic viscosity at 25 ℃ (mPa·s)	Relative permittivity	Suppliers
Hexadecane	99%	3.039[1]	2.02[2]	Shanghai Aichun
Squalane	2,6,10,15,19,23-Hexamethyltetracosane	28.257[3]	1.92/2.09[4]	Shanghai Aichun
PAO6	Poly Alpha Olefin, SpectraSyn 6	45.79[5]	2.1[6]	Chevron Phillips Chemical
BMIMPF6	1-Butyl-3-methylimidazolium hexafluorophosphate	272.86[7]	14.0±0.7[8]	Shanghai Aichun
Silicone oil	Polydimethylsiloxane	47.5[9]	2.72[9]	Dow Corning

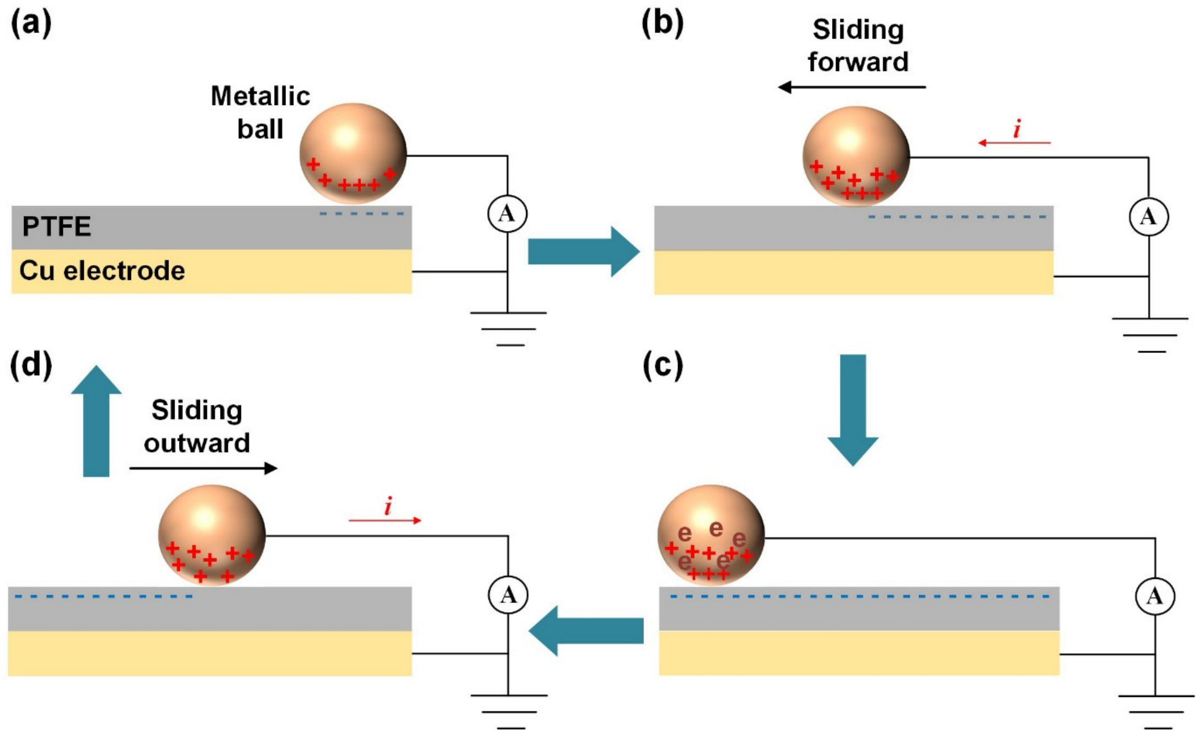

Figure S3. Schematic diagram of the working mechanism of the reciprocating sliding-mode TENG based on PTFE film and the metallic ball. (a) When the steel ball and PTFE film come into contact with each other, the steel ball is positively charged and the PTFE film is negatively charged. The net charge is zero due to the equivalent accumulation of positive and negative charges on the contact surface. (b) When the steel ball slides forward (from right to left), free electrons flow from the bottom Cu electrode to the metallic ball. (c) The metallic ball approaches the maximum displacement. (d) When the steel ball slides outward (from left to right), electrons flow back from the metallic ball to the bottom Cu electrode. The output short-circuit current exhibits a bipolar peak shape with alternating positive and negative, as shown in Figure S4c and d.

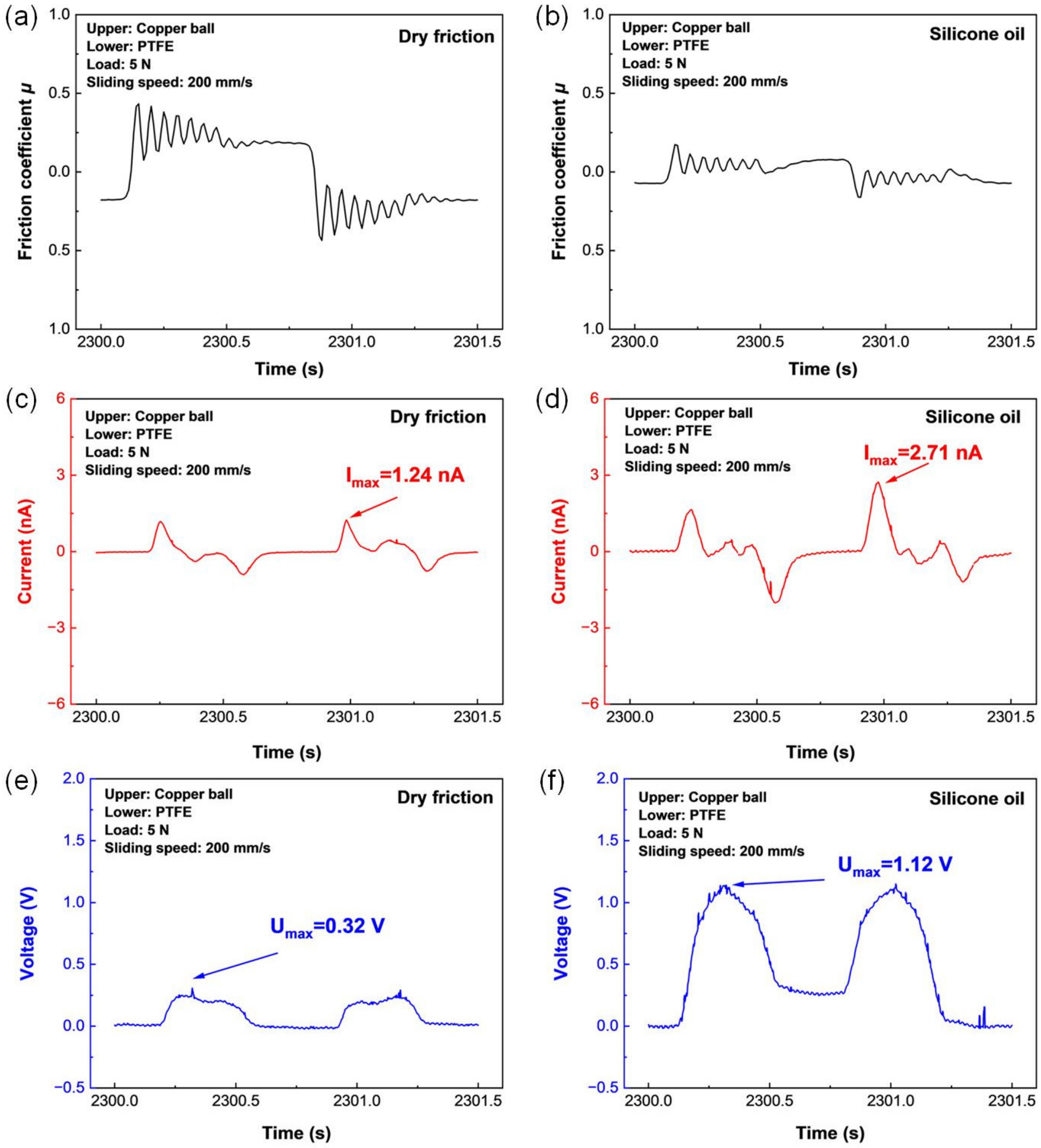

Figure S4. Typical triboelectrical performances of the PTFE film sliding against copper ball under (a, c, e) dry friction and (b, d, f) silicone oil lubrication conditions with a load of 5 N and a sliding speed of 200 mm/s in one cycle at steady stage (i.e., 2300 s). (a-b) Friction curve. (c-d) Output short-circuit current. (e-f) Output open-circuit voltage.

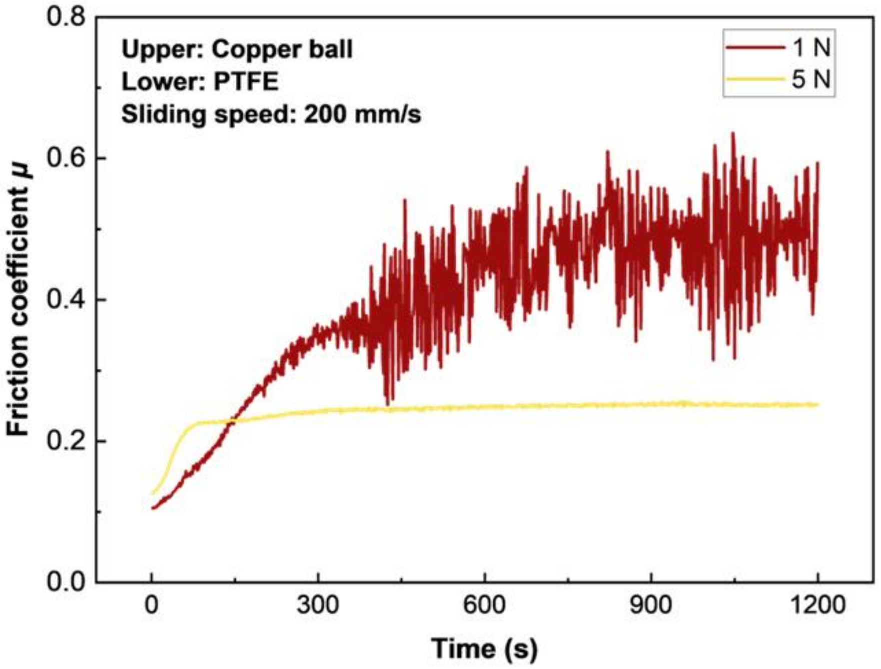

Figure S5. Friction curves of the PTFE film sliding against copper ball under dry friction condition with the load of 1 N and 5 N.

Table S3. Summary of the studies on low friction electrification mechanism of the TENGs.

No.	Tribopairs	Lubricants	Contact form	Environment	Friction coefficient	Current & Voltage	Mechanisms	Ref.
1	PTFE/ Nylon	Hexadecane	Face to face	Ambient air	0.04	15 μA<br>60 V	(i) Formation of boundary lubrication film; (ii) Hexadecane film enhanced the surface charge density	[10]
2	Steel/ PVDF	PAO 4	Ball-on-disc	Ambient air	0.06	6 nA<br>0.15 V	(i) Formation of stable lubricating oil film; (ii) Lubricant suppressed the air breakdown	[11]
3	Steel/ PTFE	Hexadecane & OLC	Face to face	Ambient air	0.048	400 nA<br>40 V	(i) "Micro-bearing" effects; (ii) Reducing the generation of PTFE transfer film and enhanced contact area	[12]
4	Graphite/ n-Si	None	AFM tip controls graphite sliding on n-Si	Ambient air	0.0039	3.4 nA<br>0.13 V	(i) Incommensurate crystalline contact; (ii) Non-equilibrium electric field caused electronic drift	[13]
5	Steel/ Hydrogen containing DLC film	None	Ball-on-disc	Nitrogen atmosphere	0.006	16 nA<br>1.6 V	(i) Low energy barrier of the hydrogen-terminated surfaces; (ii) The tribovoltaic of the rubbing interface	[14]
6	Steel/ PTFE	Silicone oil & graphene nanosheets	Ball-on-disc	Ambient air	0.008	9.24 nA<br>0.95 V	(i) Formation of graphene-rich transfer film; (ii) Channel effect of the graphene nanosheets	This work

Video S1 Video of measuring the output open-circuit voltage of the PTFE film sliding against steel ball under graphene doped silicone oil lubrication condition.

References

[1] Lal, K.; Tripathi, N.; Dubey, G.P. Densities, viscosities, and refractive indices of binary liquid mixtures of hexane, decane, hexadecane, and squalane with benzene at 298.15 K. *J. Chem. Eng. Data* **2000**, *45*, 961–964.

[2] Aralaguppi, M.I.; Aminabhavi, T.M.; Balundgi, R.H.; Joshi, S.S. Thermodynamic interactions in mixtures of bromoform with hydrocarbons. *J. Phys. Chem.* **1991**, *95*, 5299–5308.

[3] Dubey, G.P.; Sharma, M.; Oswal, S. Volumetric, transport, and acoustic properties of binary mixtures of 2-methyl-1-propanol with hexadecane and squalane at T = (298.15, 303.15, and 308.15) K: experimental results, correlation, and prediction by the ERAS model. *J. Chem. Thermodyn.* **2009**, *41*, 849–858.

[4] Bouteloup, R.; Mathieu, D. Predicting dielectric constants of pure liquids: Fragment-based kirkwood-frohlich model applicable over a wide range of polarity. *Phys. Chem. Chem. Phys.* **2019**, *21*, 11043–11057.

[5] Guimarey, M.J.G.; Comuñas, M.J.P.; López, E.R.; Amigo, A.; Fernández, J. Thermophysical properties of polyalphaolefin oil modified with nanoadditives. *J. Chem. Thermodyn.* **2019**, *131*, 192–205.

[6] Rosenkranz, A.; Martin, B.; Bettscheider, S.; Gachot, C.; Kliem, H.; Mücklich, F. Correlation between solid-solid contact ratios and lubrication regimes measured by a refined electrical resistivity circuit. *Wear* **2014**, *320*, 51–61.

[7] Goddu, B.; Tadavarthi, M.M.; Tadekoru, V.K.; Guntupalli, J.N. Density, speed of sound, and dynamic viscosity of 1-butyl-3-methylimidazolium bis(trifluormethylsulfonyl)imide/1-butyl-3-methylimidazolium hexafluorophosphate and N-methylaniline binary systems from T=298.15 to 323.15 K at 0.1 MPa. *J. Chem. Eng. Data* **2019**, *64*, 2303–2319.

[8] Huang, M.M.; Jiang, Y.; Sasisanker, P.; Driver, G.W.; Weingärtner, H. Static relative dielectric permittivities of ionic liquids at 25 °C. *J. Chem. Eng. Data* **2011**, *56*, 1494–1499.

[9] Ishikawa, T.; Yasuda, K.; Igarashi, T.; Yanabu, S.; Ueta, G.; Okabe, S. Effect of temperature on the streaming electrification characteristics of silicone oil. *IEEE T. Dielect. El. In.* **2009**, *16*, 273–280.

[10] Wang, K.Q.; Li, J.J.; Li, J.F.; Wu, C.Y.; Yi, S.; Liu, Y.F.; Luo, J.B. Hexadecane-containing sandwich structure based triboelectric nanogenerator with remarkable performance enhancement. *Nano Energy* **2021**, *87*, 106198.

[11] Liu, X.; Zhang, J.J.; Zhang, L.Q.; Feng, Y.G.; Feng, M.; Luo, N.; Wang, D.A. Influence of interface liquid lubrication on triboelectrification of point contact friction pair. *Tribol. Int.* **2022**, *165*, 107323.

[12] Guo, Y.F.; Zhang, L.Q.; Du, C.H.; Feng, Y.G.; Yang, D.; Zhang, Z.T.; Feng, M.; Wan, Y.; Wang, D.A. Onion-like carbon as nano-additive for tribological nanogenerators with enhanced output performance and stability. *Nano Energy* **2022**, *104*, 107900.

[13] Huang, X.Y.; Xiang, X.J.; Nie, J.H.; Peng, D.L.; Yang, F.W.; Wu, Z.H.; Jiang, H.Y.; Xu, Z.P.; Zheng, Q.S. Microscale schottky superlubric generator with high direct-current density and ultralong life. *Nat. Commun.* **2021**, *12*, 2268.

[14] Zhang, L.Q.; Cai, H.F.; Xu, L.; Ji, L.; Wang, D.A.; Zheng, Y.B.; Feng, Y.G.; Sui, X.D.; Guo, Y.F.; Guo, W.L.; Zhou, F.; Liu, W.M.; Wang, Z.L. Macro-superlubric triboelectric nanogenerator based on tribovoltaic effect. *Matter* **2022**, *5*, 1532–1546.
